# Clinical effect of intravitreal ranibizumab and laser photocoagulation on diabetic macular edema: a meta-analysis

**DOI:** 10.3389/fendo.2025.1608456

**Published:** 2026-09-16

**Authors:** Linyu Hu, Yan Li, Qingmin Ma, Jianmin Wang

**Affiliations:** Department of Ophthalmology, Hebei Provincial People’s Hospital, Shijiazhuang, China

**Keywords:** intravitreal ranibizumab, laser photocoagulation, diabetic macular edema, acuity, meta-analysis

## Abstract

**Objective:**

To systematically evaluate the clinical effect of intravitreal ranibizumab (IVR) and laser photocoagulation on diabetic macular edema (DME).

**Method:**

A systematic search was conducted of five English databases from the establishment of each database to 31 January 2024, and a meta-analysis was performed using Review Manager 5.3 software.

**Result:**

A total of 1,040 patients were included across 10 articles, where 531 patients had been treated with IVR and 509 with laser photocoagulation. The results of the meta-analysis showed that visual acuity improvement in the IVR group was better than in the laser photocoagulation group. The difference was statistically significant (best corrected visual acuity [BCVA], Mean Difference (MD) = 5.69, 95% confidence interval [CI]: 4.51, 6.86, *P* < 0.001). There was no significant difference in BCVA based on Logarithm of the Minimum Angle of Resolution (logMAR) value (MD = −0.07, 95%CI: −0.15, −0.01, *P* = 0.07). The improvement of macular edema in IVR was better than in the laser photocoagulation group (central macular thickness, MD = −30.93, 95% CI: −35.18, −26.68, *P* < 0.001). There were more adverse reaction events in the IVR group than in the laser photocoagulation group, and the difference was statistically significant (odds ratio = 8.82, 95% CI: 3.06, 25.43, *P* < 0.001).

**Conclusion:**

Intravitreal ranibizumab remains superior to laser photocoagulation in improving vision and reducing macular edema in DME, albeit with higher adverse events. These findings support IVR’s continued relevance in contemporary practice, particularly where newer anti-vascular endothelial growth factor agents are unavailable.

## 1 Introduction

Diabetic retinopathy (DR) refers to patients with diabetes who suffer from hyperglycemia, leading to microvascular lesions in the tissues and organs of the entire body, necrosis of the pericytes of capillaries, the thinning of endothelial cells, impaired internal barrier function and the leakage of liquid components from blood vessels into tissues. Retinopathy and diabetic dysfunction can cause lesions in the tissues of the eye, and retinopathy is the most serious complication of irreversible blindness in diabetic eye disease. Among these complications, diabetic macular edema (DME) is the primary cause of visual and impairment in diabetic patients (1).

There are a variety of treatment methods for diabetic macular edema. Since the late 1980s, macular laser grid photocoagulation has been used as the preferred treatment for DME, and the Early Treatment Diabetic Retinopathy Study (ETDRS) showed that Gesan-like laser photocoagulation could reduce the risk of vision loss by 50% (2). However, this method can only help to alleviate further vision loss and stabilize existing vision, but cannot restore vision that has already been lost (3). In addition, other treatment methods such as intravitreal injection of triamcinolone, intra-eye glucocorticoid implantation devices (e.g. the dexamethasone implantation device, Ozurdex, and the triamcinolone implantation device I-vation), protein kinase C inhibitors and tumour necrosis factor, etc.

In recent years, studies have found that the development of DME is closely related to vascular endothelial growth factor (VEGF), which can regulate vascular permeability, while its inhibition can effectively treat diabetic macular edema. Existing VEGF inhibitors include bevacizumab, ranibizumab, albocept and combocept. Among them, ranibizumab is the first anti-VEGF drug approved by the Food and Drug Administration (FDA) for the treatment of DME. It is derived from the Fab fragment of bevacizumab, the molecular weight of which is only 1/3 of that of bevacizumab, with better diffusion and a lower possibility of causing an immune response among inflammatory cells (4). Compared with laser photocoagulation, which had been the dominant therapy in the past, the advantages of anti-VEGF drugs have become more obvious (5).

With the wide application of anti-VEGF drugs, in recent years, whether ranibizumab or laser photocoagulation, or ranibizumab combined with laser photocoagulation, is more suitable for treating DME, has become a focus of debate among ophthalmic clinicians. A large number of clinical trials targeting ranibizumab and laser therapy for DME have also been conducted at home and abroad. Through a systematic review and meta-analysis, this study comprehensively collected the currently published relevant literature to compare the clinical efficacy and safety of intravitreal ranibizumab (IVR) and laser photocoagulation in the treatment of DME to provide a basis for guiding clinical treatment. While recent advancements have introduced aflibercept and faricimab, ranibizumab persists as a cornerstone therapy due to its biosimilar availability and inclusion in global guidelines. This meta-analysis re-evaluates IVR vs laser photocoagulation, not to challenge prior landmark trials, but to consolidate newer evidence (2011–2023) and reaffirm its utility in diverse clinical settings.

## 2 Data and methods

### 2.1 Literature retrieval strategy

Following the Preferred Reporting Items for Systematic Reviews and Meta-Analyses (PRISMA) guidance manual, five English databases (PubMed, the Cochrane Library, EMBASE, Web of Science and the National Library of Medicine) were systematically searched. The search period extended from the establishment of each of the databases to 31 January 2024. The search was conducted using a combination of subject terms and free words, and the references included in the study and related reviews were traced. The search formula was as follows: (1) ‘diabetic macular edema’ OR ‘diabetic macular edema’ AND ‘treatment’; (2) ‘diabetic macular edema’ OR ‘diabetic macular edema’ AND ‘lucentis’ OR ‘ranibizumab’; (3) ‘diabetic’ AND ‘macular edema’ OR ‘diabetic macular edema’, ‘photocoagulation’ OR ‘laser’. When searching, the researchers first used ‘title/abstract’ as the qualifier; if the number of retrieved documents was too small, this was changed to ‘full text’ for searching.

### 2.2 Criteria for inclusion and exclusion of references

Inclusion criteria:

1. Study type: randomized controlled trial (RCT);
2. Study participants: patients with diabetic macular edema, regardless of diabetes type, aged above 18 years old, gender.

Diagnostic criteria:

1. Clinically significant macular edema (CSME), as defined by the ETDRS criteria (6, 7):
  –retinal thickening within 500 micrometers of the center of the macula
  –hard exudates within 500 micrometers of the center of the macula if associated with adjacent retinal thickening
  –a zone of retinal thickening of at least one disc area in size, any part of which is within one disc diameter of the center of the macula.
2. Auxiliary examination: fundus fluorescein angiography (FFA) or optical coherence tomography (OCT).
3. Intervention measures: IVR alone or retinal laser photocoagulation alone, specifically including panretinal photocoagulation (PRP) and grid laser photocoagulation; patients who received 2 or more treatments within 6 months were excluded.
4. Outcome indicators:

Primary outcomes: All visual acuity measurements were converted to best corrected visual Acuity (BCVA). The criterion for visual acuity improvement was an increase of 3 or more lines on the ETDRS eye chart, as defined by the Early Treatment Diabetic Retinopathy Study Group. The criterion for unchanged vision was ETDRS vision remaining the same or changing within 3 lines. Visual impairment was defined as a decrease of 3 or more lines in ETDRS vision.

Changes in macular edema: central macular thickness (CMT) was measured, and a clinical fundus examination, FFA or OCT examination was performed.

Secondary outcomes: Adverse reactions – included conjunctival hemorrhage, eye pain, eye congestion, increased intraocular pressure, hypertension, nausea, hypoglycemia and congestive heart failure.

Exclusion criteria:

1. Macular edema caused by other reasons;
2. focal laser photocoagulation in the macular area was excluded, as it is typically used for specific macular lesions rather than as a primary treatment for DME;
3. patients with severe cataract, vitreous turbidity and glaucoma;
4. patients with incomplete data, or who were unable to provide original data;
5. repeated published studies;
6. reviews, cases, and other studies;
7. patients who had received treatment for DME, panretinal photocoagulation, or who had undergone any major ocular surgery within 3–4 months prior to the current study.

### 2.3 Literature screening and data extraction

The literature was screened independently by two researchers. The titles and abstracts were initially screened, then the full text was read according to the inclusion and exclusion criteria for secondary screening. When opinions were inconsistent, the views of the third researcher were solicited and discussed to reach a unified opinion. After the literature screening was completed, data were extracted independently by two researchers, including the basic characteristics of the included study: first author, publication year, publication country, sample size, intervention measures, intervention time, outcome indicators and measurement time.

### 2.4 Risk of bias assessment of included studies

The results were cross-checked by two researchers who independently assessed the risk of bias being present in the studies. The risk of bias in RCTs was evaluated using the RCT risk assessment tool recommended in the Cochrane Manual 5.1.0 (8). The evaluation contents include: (1) random sequence generation; (2) allocation concealment; (3) blinding of participants and personnel; (4) blinding of the outcome assessment; (5) incomplete outcome data; (6) selective reporting; (7) other bias. Each criterion was evaluated using ‘low risk bias’, ‘high risk bias’ and ‘unclear’. The literature quality was divided into A, B and C (three) grades, where A fully met the standard, B partially met the standard, and C did not meet the standard. If there was disagreement about the evaluation results, the third researcher was consulted.

### 2.5 Statistical analysis method

Meta-analysis was performed using the RevMan 5.3 (The Nordic Cochrane Centre, Copenhagen, Denmark). An odds ratio (OR) was used as the effect indicator, and weighted mean difference (WMD) was used as the effect indicator for measurement data. All effect sizes were calculated with point estimates and a 95% confidence interval (CI). A heterogeneity test was used to determine the heterogeneity size, *I*^2^< 50% or *P* > 0.1, and the included literature was considered to be homogeneous. A fixed effect model (Mantel–Haenszel) was used for analysis. If *I*^2^>50% or *P* ≤ 0.1, a degree of heterogeneity among the included studies was assumed. A random effects model (DerSimonian–Laird) was used for analysis. The test level of the meta-analysis was set as α = 0.05.

## 3 Results

### 3.1 Literature search results

A total of 346 relevant literature studies were retrieved for the present research. After systematic screening, duplicate literature and papers that did not meet the inclusion criteria were excluded. Finally, 10 studies that met the criteria (9–18) were included for the meta-analysis and systematic review. A flow chart of the literature retrieval and screening process is shown in **Figure 1**.

**Figure 1:**
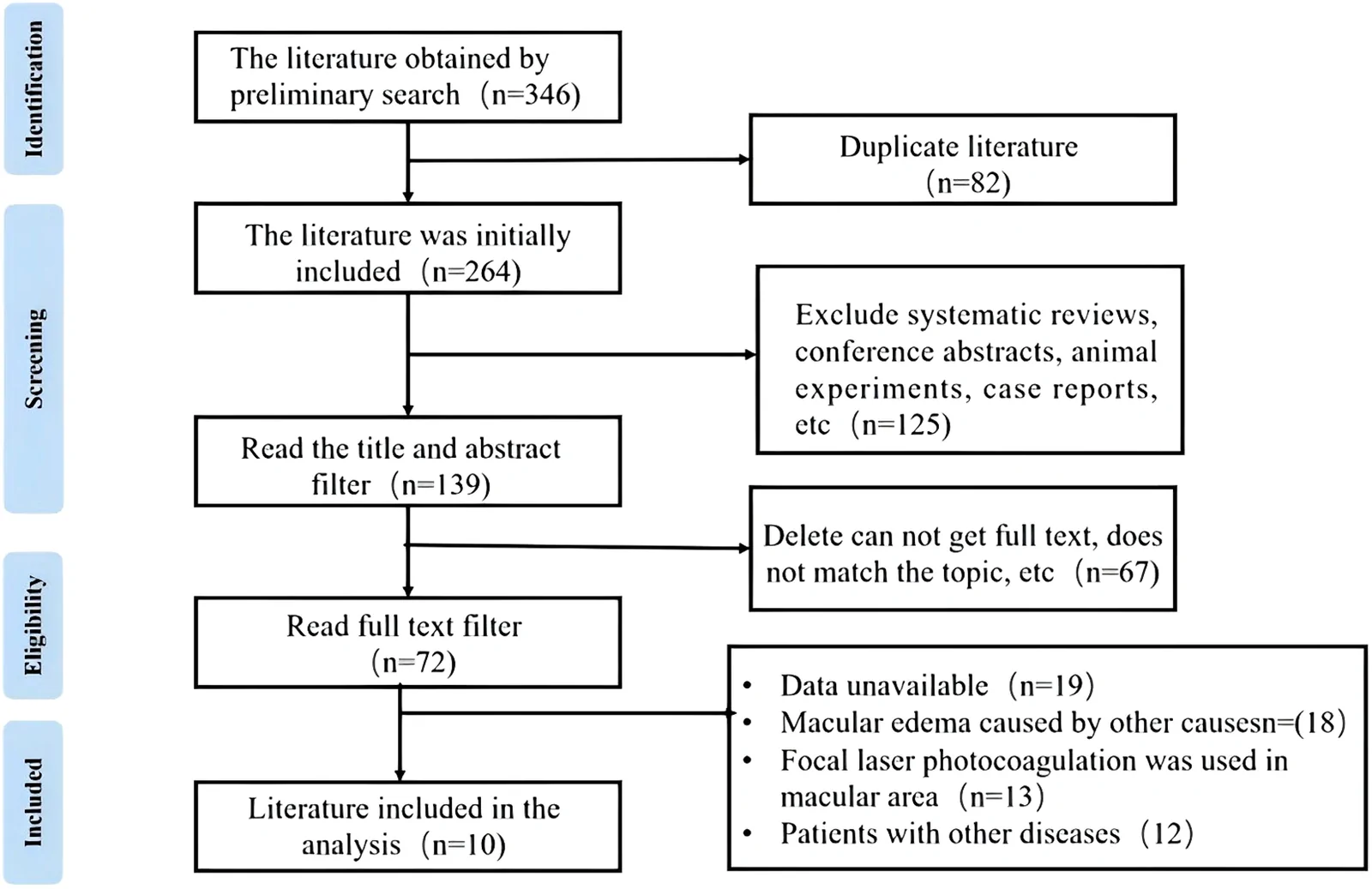
Literature screening flow chart.

### 3.2 Basic characteristics of the included studies and results of the risk of bias assessment

The basic characteristics of the included literature are shown in **Table 1**. A total of 1,040 patients were included across 10 articles, where 531 patients had been treated with IVR and 509 with laser photocoagulation. The retrieved studies were published between 2011 and 2023, except for two studies (10, 12) that had a follow-up period of 6 months; the remaining studies had follow-up periods of 12 months.

**Table 1:** Basic characteristics of included studies and literature quality evaluation levels.

Included literature	Year of publication	Publication area	Sample size		Age		Gender (M/F)		Number of eyes/pairs		Dose	Follow-up time	Outcome
			IVR	Laser	IVR	Laser	IVR	Laser	IVR	Laser	IVR		
Buyru (9)	2018	Turkey	27	24	64.7 ± 0.87	65.33 ± 11.82	18/9	14/10	27	24	0.5mg	12 months	1, 2
Kumar (10)	2019	France	15	15	53.40 ± 5.32	57.93 ± 7.21	8/7	7/8	15	15	3 injections 0.5 mg	6 months	1, 2
Feng (11)	2023	China	49	49	59.42 ± 10.10	57.06 ± 7.79	24/25	17/28	49	45	1.25 mg/0.05 mL per injection	12 months	1, 2
Terashima (12)	2019	Japan	24	22	67.9 ± 8.9	67.9 ± 8.9	10/14	10/12	24	22	/	6 months	1, 2
Cao (13)	2019	China	20	20	66.90 ± 6.52	66.90 ± 6.52	12/8	13/7	20	20	0.5mg/0.05ml	12 months	1, 2
Do (14)	2012	USA	44	44	–	–	–	–	42	42	0.5mg	12 months	1, 2
Berger (15)	2013	Canada	81	82	62 ± 9.8	62 ± 9.8	–	–	81	82	0.54mg	12 months	1, 2
Comyn (16)	2014	UK	22	11	64.9	67.4	15/7	6/5	22	11	0.5mg/0.05ml	12 months	1, 2
Mitchell (17)	2011	USA	116	111	62.9 ± 9.29	64.0 ± 8.15	73/43	58/53	116	111	0.5mg	12 months	1, 2, 3
Ohji (18)	2012	East Asia	133	131	60.7	61.5	–	–	133	131	0.5mg	12 months	1, 2, 3

1=change of best corrected visual acuity (BCVA); 2=central macular thickness (CMT); 3=Adverse Events.

Regarding patient characteristics, the age of enrolled patients ranged from 53.40 ± 5.32 years to 67.9 ± 8.9 years, with the majority of studies including patients with a mean age over 60 years, consistent with the typical onset of diabetic macular edema (DME) in middle-aged and elderly diabetic patients. The gender distribution was generally balanced across studies, although some studies did not report detailed gender data.

Specific details of the interventions are as follows:

- IVR Group: Most studies administered ranibizumab via intravitreal injection at a dose of 0.5 mg per eye. Some studies, such as Feng et al., used a dose of 1.25 mg/0.05 mL, while Berger et al. used 0.54 mg. The injection frequency was typically monthly or pro re nata (3+PRN, as needed) (3+PRN), with a treatment duration ranging from 6 to 12 months.
- Laser Photocoagulation Group: The intervention methods included panretinal photocoagulation (PRP) and grid laser photocoagulation. None were combined with anti-VEGF agents. Laser parameters and the number of treatment sessions varied according to the respective study protocols, with follow-up times consistent with the IVR group.

Regarding the use of biosimilars, none of the studies included in this meta-analysis explicitly reported the use of ranibizumab biosimilars. All interventions referred to the originator product ranibizumab (Lucentis^®^).

In terms of the risk of bias assessment, eight of the ten studies were rated as Grade A (low risk of bias), and two were rated as Grade B (moderate risk of bias), primarily due to unclear reporting of allocation concealment or blinding procedures. Overall, the methodological quality was high, supporting the reliability of the findings. For details, see **Table 2**.

**Table 2:** Results of risk of bias assessment of included studies.

Included literature	Random sequence generation	Allocation concealment	Blinding of participants and personnel	Blinding of outcome assessment	Incomplete outcome data	Selective reporting	Other bias	Level of evidence
Buyru (9)	Low	Low	Low	Low	Low	Low	Low	A
Kumar (10)	Low	Low	Low	Low	Low	Low	Low	A
Feng (11)	Low	Low	Low	Low	Low	Low	Low	A
Terashima (12)	Low	Low	Low	Low	Low	Low	Low	A
Cao (13)	Low	Low	Low	Low	Low	Low	Low	A
Do (14)	Low	Low	Not clear	Not clear	Low	Low	Low	B
Berger (15)	Low	Low	Low	Low	Low	Low	Low	A
Comyn (16)	Low	Low	Low	Low	Low	Low	Low	A
Mitchell (17)	Low	Not clear	Not clear	Low	Low	Low	Low	B
Ohji (18)	Low	Low	Low	Low	Low	Low	Low	A

### 3.3 Meta-analysis results

#### 3.3.1 Visual improvement

Data before and after BCVA were reported in 10 of the included papers. Five studies reported Logarithm of the Minimum Angle of Resolution (logMAR) vision data, and the remaining 5 reported converted BCVA data. Accordingly, the meta-analysis was conducted separately.

(1) Logarithm of the Minimum Angle of Resolution

The logMAR values of IVR and laser photocoagulation before and after surgery were reported in 5 articles. In this paper, changes in logMAR before and after surgery were analyzed to represent improvements in visual acuity. The analysis results showed that there was heterogeneity among the studies (*I^2^* = 58.0%). A meta-analysis using a random effects model was performed (see **Figure 2**). The results showed that the visual acuity improvement in the IVR group was significantly higher than in the laser photocoagulation group. However, the difference was not statistically significant (Mean Difference [MD] = −0.07, 95% CI: −0.15, −0.01, *P* = 0.07).

**Figure 2:**
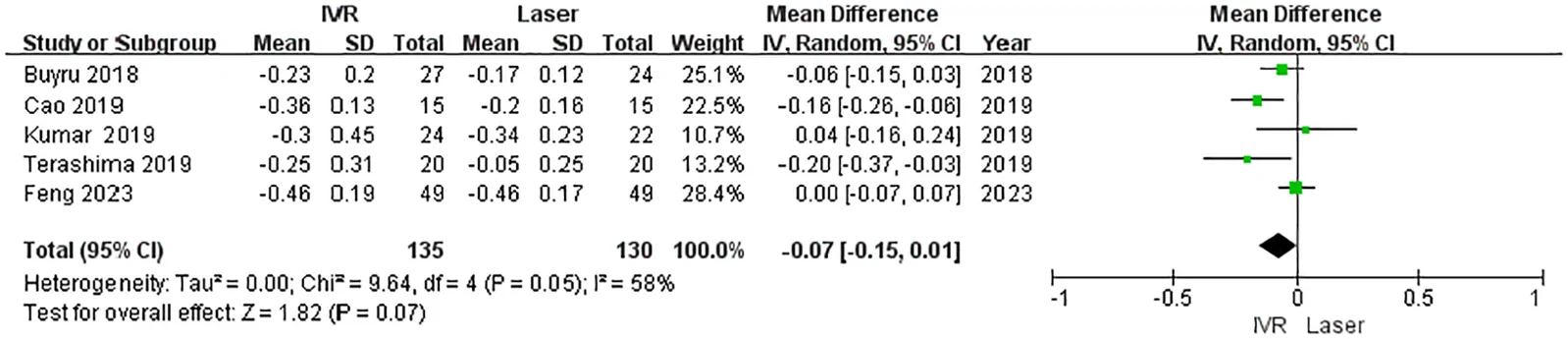
Forest map of logMAR in IVR group and laser photocoagulation group.

Best corrected visual acuity

The BCVA values before and after surgery for IVR and laser photocoagulation were reported in 5 articles. In this paper, changes in BCVA before and after surgery were analyzed to represent improvements in visual acuity. The analysis results indicated no heterogeneity among the studies (*I^2^* = 0%). A meta-analysis using a fixed-effect model was performed (see **Figure 3**). The results showed that visual acuity improvement in the IVR group was more obvious compared with the laser photocoagulation group. The difference was statistically significant (MD = 5.69, 95% CI: 4.51, 6.86, *P* < 0.001).

**Figure 3:**
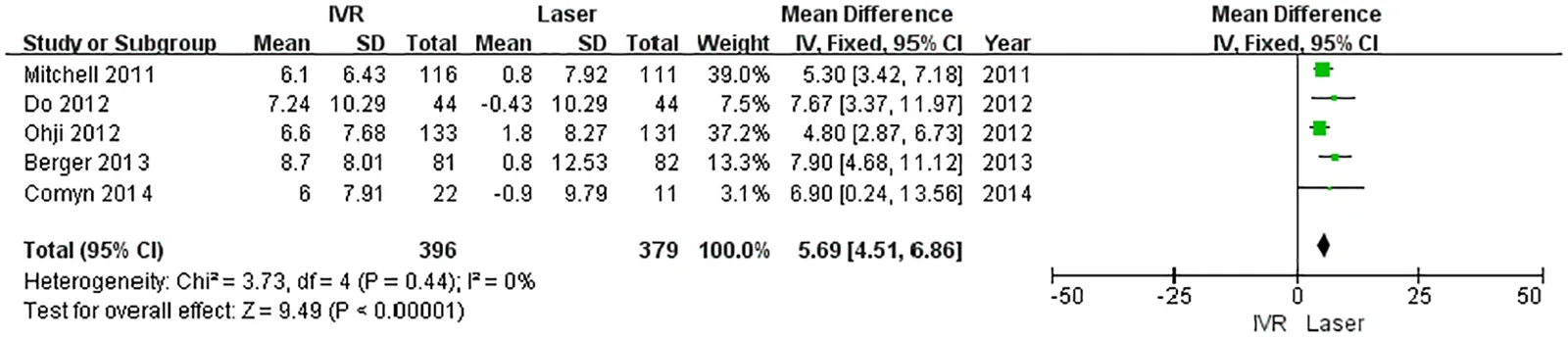
Forest map of BCVA in IVR group and laser photocoagulation group.

#### 3.3.2 Changes in macular edema

The CMT values before and after surgery of IVR and laser photocoagulation were reported in the 10 included studies. In this paper, the change in values before and after surgery was analyzed to represent changes in macular edema. The analysis results indicated a low degree of heterogeneity among the studies (*I^2^* = 48.0%). A meta-analysis using a fixed-effect model was performed (see **Figure 4**). The results showed that macular edema improved better in the IVR group than in the laser photocoagulation group. The difference was statistically significant (MD = −30.93, 95% CI: −35.18, −26.68, *P* < 0.001).

**Figure 4:**
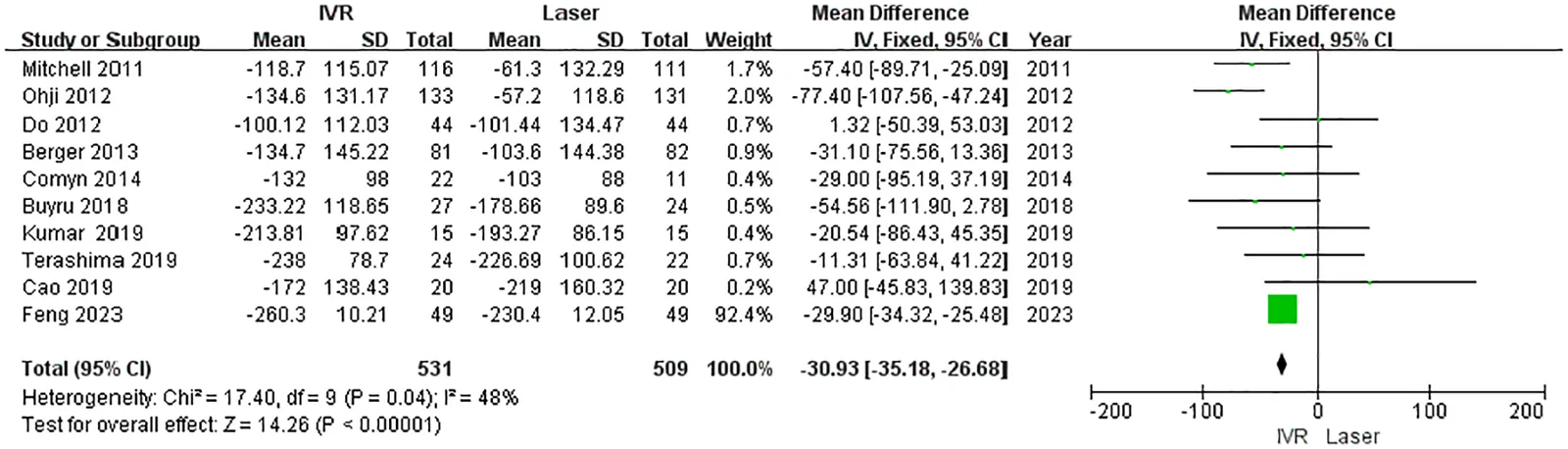
Forest map of CMT in IVR group and laser photocoagulation group.

#### 3.3.3 Adverse event

Two studies (17, 18) reported the occurrence of adverse events such as Subconjunctival hemorrhage, elevated intraocular pressure, endophthalmitis, retinal detachment. The analysis results showed that there was no heterogeneity among the studies (*I^2^* = 0%). A meta-analysis was performed using the fixed effect model (see **Figure 5**). The results showed that there were more adverse events in the IVR group than in the laser photocoagulation group. The difference was statistically significant (OR = 8.82, 95% CI: 3.06, 25.43, *P* < 0.001).

**Figure 5:**
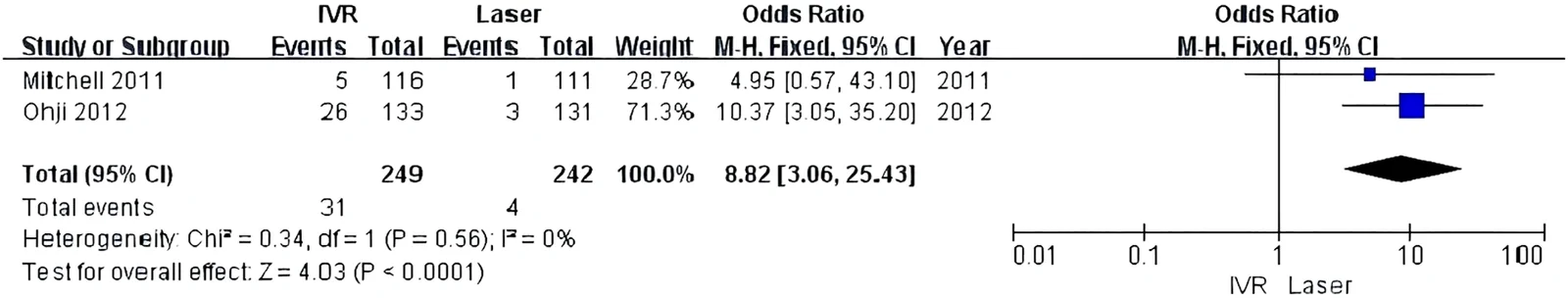
Forest map of adverse event in IVR group and laser photocoagulation group.

#### 3.3.4 Publication bias

Publication bias analysis was performed on logMAR, BCVA and CMT. As shown in **Figure 6**, the CMT funnel plot was obviously asymmetrical, suggesting the possible existence of publication bias.

**Figure 6:**
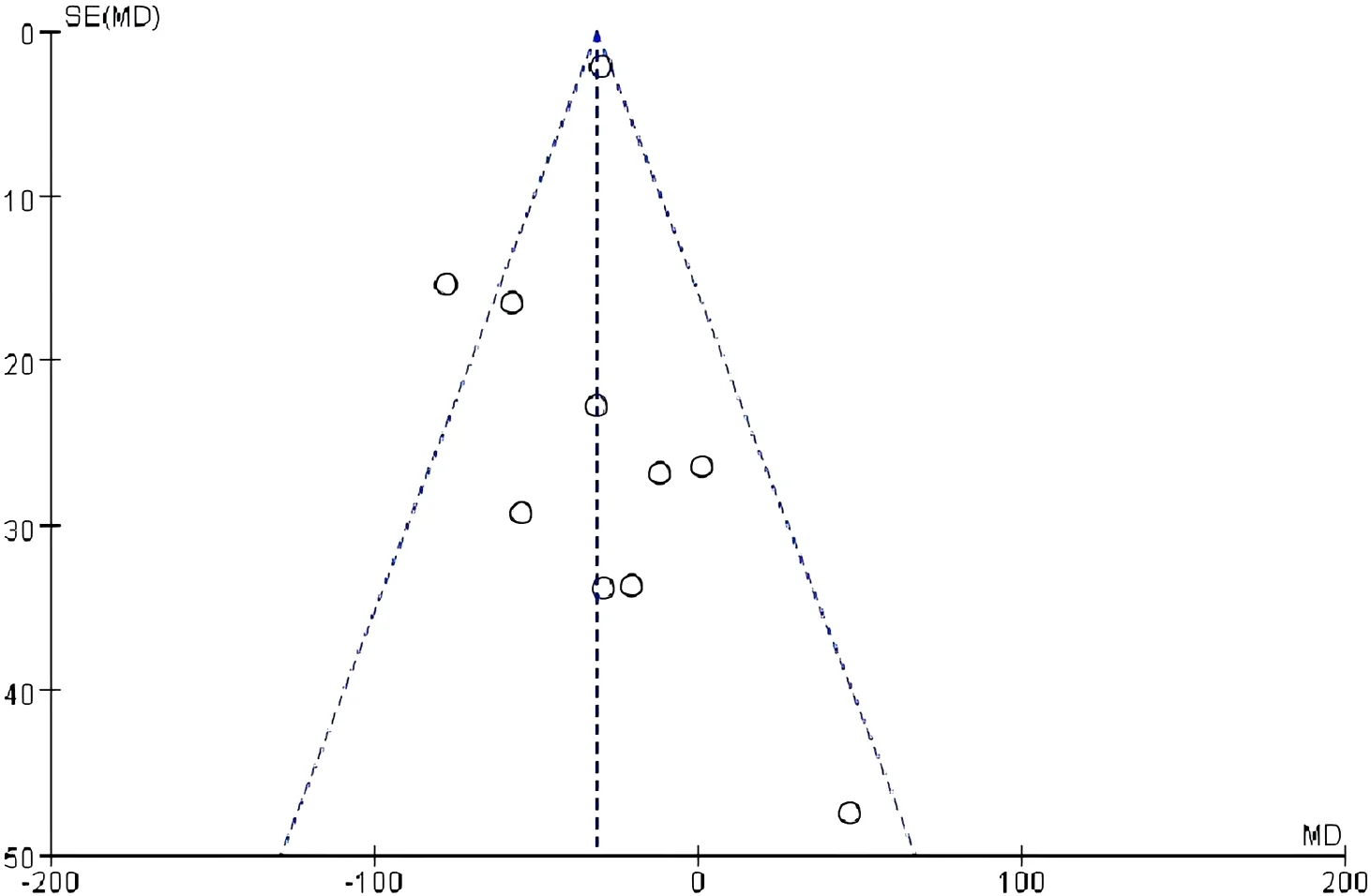
Analysis of publication bias (CMT).

## 4 Discussion

The clinical effects of IVR and laser photocoagulation on DME were compared. Through the method of a systematic review and meta-analysis, the results of the published literature were integrated. All 10 studies included in the meta-analysis were RCTs. Allocation hiding was implemented in all 10 studies. The participants and researchers were blinded in 9 studies, and the outcome measures were blinded in 9 studies. The overall quality level of the included studies was high, and the research results indicated a degree of reliability. The results of the meta-analysis showed that compared with laser photocoagulation therapy, IVR alone could significantly improve the visual acuity level and alleviate macular edema in patients with diabetic macular edema, but the treatment may cause adverse reactions.

The results of this study indicate that improvement in visual acuity for patients with DME treated with IVR was significantly better when compared with laser photocoagulation treatment. There was no significant difference in visual acuity improvement based on the logMAR value. This result is consistent with research conducted by Liao et al. (19), which included 1,749 affected eyes (IVR, 394; laser photocoagulation, 713) and compared the effect of IVR and laser photocoagulation on DME. The improvement degree of BCVA was significantly better in the IVR group after surgery (WMD = 5.65, 95% CI: 4.44-6.87, *P* < 0.01). The possible mechanisms for the above results are as follows: the occurrence and development of DME is related to VEGF, which can directly induce inflammation, promote endothelial cell mitosis and lead to neovascularization (20, 21). At the same time, VEGF can indirectly promote angiogenesis as a survival factor for vascular endothelial cells (22). It can also promote vascular endothelial window opening or destroy endothelial cell connections by increasing the phosphorylation of connexin (23). As a result, vascular permeability is increased, resulting in the extravasation of liquid and plasma components into the retina, which thickens and results in edema (24). Ranibizumab is the first anti-VEGF drug approved by the FDA for the treatment of DME, and in recent years, IVR has shown good clinical effect on DME (25). Due to the small molecular weight of ranibizumab and its strong penetration and permeability, the bioavailability of the drug is high when injected into the vitreous body, which can be more than 50% (26). At the same time, it can also reduce the ocular vascular permeability, reduce the expression of inflammatory factors, strengthen the role of the blood–retinal barrier, reduce the formation of new blood vessels and help improve the BCVA (27).

The results of this study indicate that macular edema improved better in patients with DME who were treated with IVR compared with those treated with laser photocoagulation. The changes in macular edema were measured by preoperative and postoperative CMT values, which were examined by OCT and followed up for 6 months or more. In the late 1980s, macular edema was defined as (28): (1) more severe, rigid exudation than DR research standard number 3; (2) retinal thickening caused by fluid accumulation in the diameter of one optic disc in the central macular region that which was twice the diameter of the central vein. Therefore, the use of CMT can be more intuitive to indicate the degree of macular edema. In this meta-analysis study, the changes of CMT before and after surgery were included, which can explain the improvement of macular edema in patients with DME. Diabetic macular edema can have a significant influence on the quality of daily life of patients due to varying degrees of damage to macular central visual function. Diabetic retinopathy can occur at any time. This study indicates that the degree of improvement in macular edema after 6 or 12 months of treatment in the IVR group was better than in the laser photocoagulation group, and the difference was statistically significant.

Clinically, fundus laser treatment is mainly used for DME. Laser photocoagulation causes retinal damage in the outer layer of the retinal pigment epithelium, effectively promotes the proliferation of venous endothelium and retinal microvessels, facilitates the involvement of the retinal pigment epithelium in the repair of vascular endothelial cells, enhances the transport function of the retinal pigment epithelium, and promotes the repair of the bloodresum,s barrier, thereby alleviating the symptoms of macular edema (21). However, conventional laser treatment often fails to achieve the desired therapeutic effect, and complete elimination of macular edema is unlikely (29). Once laser photocoagulation has improved the symptoms of macular edema, intravitreal injection of ranibizumab can accelerate the regression of new blood vessels in the affected area, thereby speeding up the resolution of macular edema, with better results than laser photocoagulation alone (30).

Ranibizumab has been applied in clinical settings for many years, and studies have shown that the long-term use of anti-VEGF drugs can affect the level of VEGF in the body, resulting in serious adverse reactions in various systemic systems (31). In this paper, statistics were employed and an analysis was conducted on the adverse events included in the literature studies. The results showed that two studies (15, 16) reported the occurrence of adverse events such as Subconjunctival hemorrhage, elevated intraocular pressure, endophthalmitis, retinal detachment. There were more adverse events in the IVR group than in the laser photocoagulation group, and the difference was statistically significant. Therefore, the safety of ranibizumab in patients with DME remains to be determined.

This paper has the following limitations: (1) The number of studies included and the number of patients observed were small, and the number of studies included on individual indicators, such as adverse reactions, was insufficient; as such, the results require further study. (2) The patient’s own lens condition and the evaluation of postoperative intraocular hypertension were ignored at the time of inclusion. (3) The difference in visual acuity improvement based on the logMAR value was not statistically significant between the two groups. This was due to the limitation of the small number of included studies, and may also have been caused by confounding factors, such as the course of diabetes mellitus, laser photocoagulation parameters, injection interval, and the treatment patients received prior to the study.

A critical analysis of the included studies revealed several limitations that need to be addressed in future research. The short follow-up periods in most studies limited the assessment of long-term efficacy and safety. Additionally, the small sample sizes in some of the studies may limit the generalizability of the findings. Furthermore, the heterogeneity in study designs, including variations in laser photocoagulation parameters and injection intervals, introduced potential biases and complicated the interpretation of results.

There are many therapeutic methods for treating diabetic macular edema, and laser photocoagulation has always been used in clinical treatment as the most effective treatment plan. However, in recent years, with continuing drug research, the efficacy of ranibizumab, which is an anti-VEGF, has been gradually recognized. According to clinical trials currently being conducted, during the 6-month follow-up period, the efficacy of ranibizumab was significant. However, due to the short follow-up time and small number of study cases, the efficacy and safety of ranibizumab and laser photocoagulation still require further proof via large clinical trials. For upcoming clinical trials, the follow-up time should be extended to ensure further observation. In addition, the dose of ranibizumab, the time of injection and the interval between laser photocoagulation treatments must be further tested.

## Data Availability

The original contributions presented in the study are included in the article/Supplementary Material. Further inquiries can be directed to the corresponding author.
